# DPXception: a lightweight CNN for image-based date palm species classification

**DOI:** 10.3389/fpls.2023.1281724

**Published:** 2024-01-09

**Authors:** Mejdl Safran, Waleed Alrajhi, Sultan Alfarhood

**Affiliations:** Department of Computer Science, College of Computer and Information Sciences, King Saud University, Riyadh, Saudi Arabia

**Keywords:** date palm, image classification, CNN, Xception, transfer learning, real-time application

## Abstract

**Introduction:**

Date palm species classification is important for various agricultural and economic purposes, but it is challenging to perform based on images of date palms alone. Existing methods rely on fruit characteristics, which may not be always visible or present. In this study, we introduce a new dataset and a new model for image-based date palm species classification.

**Methods:**

Our dataset consists of 2358 images of four common and valuable date palm species (Barhi, Sukkari, Ikhlas, and Saqi), which we collected ourselves. We also applied data augmentation techniques to increase the size and diversity of our dataset. Our model, called DPXception (Date Palm Xception), is a lightweight and efficient CNN architecture that we trained and fine-tuned on our dataset. Unlike the original Xception model, our DPXception model utilizes only the first 100 layers of the Xception model for feature extraction (Adapted Xception), making it more lightweight and efficient. We also applied normalization prior to adapted Xception and reduced the model dimensionality by adding an extra global average pooling layer after feature extraction by adapted Xception.

**Results and discussion:**

We compared the performance of our model with seven well-known models: Xception, ResNet50, ResNet50V2, InceptionV3, DenseNet201, EfficientNetB4, and EfficientNetV2-S. Our model achieved the highest accuracy (92.9%) and F1-score (93%) among the models, as well as the lowest inference time (0.0513 seconds). We also developed an Android smartphone application that uses our model to classify date palm species from images captured by the smartphone’s camera in real time. To the best of our knowledge, this is the first work to provide a public dataset of date palm images and to demonstrate a robust and practical image-based date palm species classification method. This work will open new research directions for more advanced date palm analysis tasks such as gender classification and age estimation.

## 1 Introduction

Date palms are a vital crop for many regions of the world, especially in arid and semi-arid regions of the Middle East and North Africa (Chao and Krueger, 2007; Rathore et al., 2020). They provide food, income, and livelihood for millions of people and have important cultural and religious significance. However, not all date palms are the same. Different species have different features such as fruit size, shape, color, texture, taste, sugar content, yield, and resistance to biotic and abiotic stresses (Al-Shahib and Marshall, 2003). These characteristics determine the quality and market value of date fruits and the suitability of date palm varieties for different environmental conditions and consumer preferences (Farooq et al., 2021). Therefore, accurate and efficient identification of date palm varieties is essential for various applications, such as breeding, management, conservation, and trade. However, date palm trees are a diverse group of plants that can be hard to tell apart by the untrained eye (Zaid and de Wet, 2002). A reliable way to identify date palm species is by using DNA tests that can tell the difference between species based on their genetic code (Al-Khalifah and Askari, 2003; Awan et al., 2017; Mahdy and El-Sharabasy, 2021; Rahman et al., 2022). However, this method is expensive, complicated, and requires specialized equipment and laboratory facilities. Alternatively, the most common way to identify date palm species is by looking at their fruits and comparing their shape, size, color, texture, and taste (Kamal-Eldin and Ghnimi, 2018). However, this method has many drawbacks: it needs experts and manual work; it takes a lot of time and effort; it can be inaccurate and subjective; and it cannot be used when the fruits are not present or visible. Consequently, there is a growing need for an efficient (real-time) and automated date palm species classification system.

**Figure 1:**
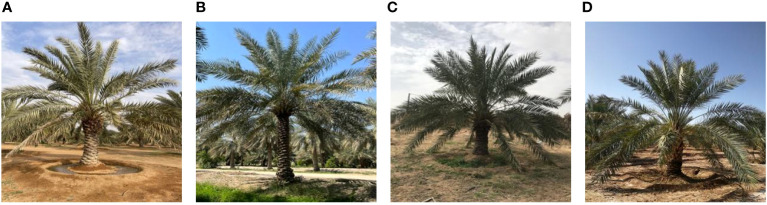
The four date palm species are: **(A)** Barhi, **(B)** Ikhlas, **(C)** Saqi, and **(D)** Sukkari.

In recent years, deep learning has shown remarkable success in image classification tasks in plant species identification and other domains (Kussul et al., 2017; He et al., 2018; Kamilaris and Prenafeta-Boldú, 2018; Li et al., 2018; Zhang et al., 2021; Bouguettaya et al., 2022). As a result, deep learning methods have emerged as a promising alternative for date palm variety identification (Haidar et al., 2012; Altaheri et al., 2019; Nasiri et al., 2019; Albarrak et al., 2022; Jintasuttisak et al., 2022; Alsirhani et al., 2023; Noutfia and Ropelewska, 2023a; Noutfia and Ropelewska, 2023b). Deep learning is a branch of machine learning that uses artificial neural networks with multiple layers to learn complex features from large amounts of data (LeCun et al., 2015; Goodfellow et al., 2016). Deep learning can automatically extract relevant features from raw images without the need for manual feature engineering or domain knowledge. Deep learning methods have several advantages over traditional and molecular methods: they are fast and easy to use; they do not require expert knowledge or manual intervention; and they can work with any parts of the date palm plant (such as leaves, stems, or fruits). Recent deep learning methods have been proposed to identify date palm species based on their fruits (Haidar et al., 2012; Altaheri et al., 2019; Nasiri et al., 2019; Albarrak et al., 2022; Jintasuttisak et al., 2022; Alsirhani et al., 2023; Noutfia and Ropelewska, 2023a; Noutfia and Ropelewska, 2023b). The main drawback of these methods is that they are designed for harvesting purposes when fruits are present; however, they cannot be used to identify date palm species when the fruits are not present or visible. Moreover, these methods only aim for classification accuracy and neglect real-time applicability. In this work, we present a novel method for automatically classifying four common date palm species from their images captured by cameras or smartphones. The date palm species we consider in this study are Barhi, Sukkari, Ikhlas, and Saqi. An example of each date palm species is shown in **Figure 1**.

This article reports our work and brings in the following main contributions:

1. We introduce a novel self-collected dataset of 2358 images of four common and valuable date palm species (Barhi, Sukkari, Ikhlas, and Saqi) from different farms in Saudi Arabia.
2. We propose *DPXception*, a lightweight and efficient CNN model that is trained and fine-tuned on our dataset to classify date palm species from images. Unlike the original Xception model, our DPXception model utilizes only the first 100 layers of the Xception model for feature extraction (resulted in an adapted Xception), making it more lightweight and efficient. We further apply normalization prior to adapted Xception and reduce the model dimensionality by adding an extra global average pooling layer after feature extraction by adapted Xception to improve the accuracy and robustness of our model.
3. We evaluate our model on our dataset and compare it with seven representative CNN models trained on our dataset. Our results show that our model outperforms the others in terms of accuracy, F1-score, and inference time.
4. We develop an Android smartphone application that incorporates our model and can classify date palm species from images captured by the phone’s camera in real-time. This demonstrates the practical applicability and usefulness of our work.

The remainder of this article is organized as follows. Section 2 surveys related works on deep learning for date palm classification and its fruit classification. Section 3 discusses the proposed approach including system pipeline, dataset collection process, *DPXception* architecture, model deployment, and development process of the proposed Android application. Section 4 reports and discusses experimental results. Finally, Section 5 concludes the article and suggests future work.

## 2 Literature review

Date palm analysis is a broad field that covers various tasks such as date palm tree and fruit classification, date palm sex identification, date palm age estimation, date palm disease detection, and date palm yield prediction. These tasks have various applications in agriculture, economy, and environment. In this section, we review the existing works that are related to our work in terms of the problem, the data, the method, and the results. We divide the related works into three subsections: date palm tree classification from aerial images, date fruit classification from orchard images, and date palm sex identification from seedling images. We then demonstrate the uniqueness of our work and how we advance the state-of-the-art in comparison with the existing works.

### 2.1 Date palm tree classification from aerial images

Aerial images, obtained from satellites, drones, or other platforms, can be used for the classification and identification of date palms. This can help in mapping, monitoring, and managing date palm plantations. Several studies have applied machine learning and deep learning techniques to this problem.

Some studies have used satellite images to identify date palms and other land covers using supervised classification methods. For example, Rahnama et al. (2018) compared the performance of Sentinel and Landsat satellites using four methods: Neural Network (NN), Maximum Likelihood Classifier (MLC), Support Vector Machines (SVM), and Mahalanobis Distance Classifier (MDC). Both satellites were able to identify the date palm areas with an average overall accuracy of more than 99%. Issa et al. (2020) used a hierarchical integrated approach to produce a detailed map of date palm plantations in the Emirate of Abu Dhabi using Landsat-8 and Worldview-2 imagery. The map depicted three different categories of date palms at three different age stages: young, medium, and mature. The map was used as input to a remote sensing-based biomass estimation model for the assessment of the above-ground biomass and carbon sequestered by date palms. Date palms were mapped with an overall accuracy of 94.5%. Another study by Culman et al. (2021) used deep learning object detection to classify and locate the Phoenix palm trees in different scenes using Sentinel-2 images over the Spanish island of La Gomera. The palm tree sub-pixel classification model achieved an overall accuracy of 0.921, with a recall and precision of 0.438 and 0.522, respectively.

Some studies have used drone images to detect and locate date palms in different scenes using deep learning object detection models. For example, Jintasuttisak et al. (2022) used YOLO-V5 to detect date palms in images captured by a drone over farmlands in the Northern Emirates of the UAE. The YOLO-V5m model achieved the highest accuracy, resulting in a mean average precision (mAP) of 92.34%.

Some studies have used UAV RGB imagery to detect other types of palm trees based on their physical morphology using transfer learning models. For example, Letsoin et al. (2022) used transfer learning to detect sago palms based on their physical morphology from the UAV RGB imagery. The ResNet-50 model was the preferred base model for sago palm classifiers, with a precision of 75%, 78%, and 83% for sago flowers, sago leaves, and sago trunk, respectively.

Some studies have used multiscale and multisource VHSR images to extract date palms from aerial images using deep vision transformers. For example, Gibril et al. (2023) investigated the reliability and the efficiency of various deep vision transformers in extracting date palms from multiscale and multisource VHSR images. The deep vision transformers achieved satisfactory results in mapping date palms from the UAV images, with a mean intersection over union (mIoU) ranging from 85% to 86.3% and a mean F1-score ranging from 91.62% to 92.44%.

### 2.2 Date fruit classification from orchard images

Orchard images, obtained from cameras mounted on robots, smartphones, or other devices, can be used for the classification of date fruits according to their type, maturity, and quality. This can help in date fruit harvesting, grading, and quality assessment. Researchers have applied machine vision and deep learning techniques to this problem.

Some studies have used Convolutional Neural Network (CNN) models to classify date fruits into different varieties and maturity stages using transfer learning and data augmentation techniques. For example, Albarrak et al. (2022) proposed a deep learning-based model that can classify date fruits into 12 varieties and maturity stages using a dataset of 12,000 images. They reported an accuracy of 98.8% for variety classification and 97.6% for maturity classification.

Some researchers have also used CNN models to classify date fruits based on their surface quality, which affects the date fruit industry. For example, Almomen et al. (2023) developed a system that can classify date fruits into excellent or poor surface quality using a new image dataset of 898 date fruits. They achieved an accuracy of 97% and stated that their system can help date fruit production and quality control. Nasiri et al. (2019) proposed a method for discriminating healthy date fruits from defective ones using a CNN model based on the VGG-16 architecture. The model was trained and tested on an image dataset containing four classes: Khalal, Rutab, Tamar, and defective date fruits. The model achieved an overall classification accuracy of 96.98%.

Some studies have used CNN models to classify date fruits based on their physical attributes or morphology using smartphone cameras or other devices. For example, Neji et al. (2021) implemented three CNN models for date fruit and leaf classification using smartphone cameras: the first classifies fruit and leaf image (binary classification) achieving accuracy of 99.97%, the second classifies fruit varieties achieving accuracy of 99.82%, and the third classifies the leaves varieties achieving accuracy of 99.73%. Bindu et al. (2022) presented a CNN architecture for classifying date fruits into four different classes based on their physical attributes: Khalas, Fardh, Khunaizi, and Qash. The system achieved a validation accuracy of 97.2%. Alaskar et al. (2022) presented a CNN architecture for classifying three varieties of date fruits (Ekhlas, Nbute Sultan, and Shayshi) using a dataset of 3165 images. The results showed that CNN can discriminate date cultivars with high accuracy.

Some researchers have proposed machine vision frameworks for date fruit harvesting robots that consist of multiple classification models based on deep learning techniques. For example, Altaheri et al. (2019) proposed a machine vision framework that consists of three classification models based on deep convolutional neural networks with transfer learning and fine-tuning on pre-trained models. The models classify date fruit images according to their type, maturity, and harvesting decision using a rich image dataset of date fruit bunches in an orchard that consists of more than 8000 images of five date types in different prematurity and maturity stages. The models achieve accuracies of more than 97% with classification times of less than 36 msec for each task. Faisal et al. (2020) proposed a smart harvesting decision system that consists of three sub-systems: Dates maturity estimation system (DMES), type estimation system (DTES), and dates weight estimation system (DWES). The DMES and DTES use four DL architectures: ResNet, VGG-19, Inception-V3, and NASNet; while the DWES uses Support Vector Machine (SVM) (regression and linear). The DTES achieves a maximum performance of 99.175% accuracy; the DMES achieves a maximum performance of 99.058% accuracy; and the DWES achieves a maximum performance of 84.27%. Al-Sabaawi et al. (2021) proposed a machine vision framework for date classification in an orchard environment using pre-trained deep learning models, with ResNet-50 achieving the highest F1-score (98.14%) and accuracy (97.37%).

### 2.3 Date palm sex identification from seedling images

Seedling images, obtained from DNA markers or other sources, can be used for the identification of the sex of date palms at the seedling stage. This is a crucial problem for date growers, as only female date palms produce fruits, and male date palms are only needed for pollination. The conventional method of sex identification relies on phenotypic characteristics that appear after several years of growth, which is costly and time-consuming. Some studies have applied machine learning and deep learning techniques to this problem. For example, Naeem et al. (2023) proposed a technique for the sex identification of date palms at the seedling stage using supervised machine learning techniques, with the Support Vector Machine (SVM) algorithm achieving 97% accuracy.

### 2.4 Discussion

We herein discuss how our work differs from the existing related works in terms of the problem, the data, the method, and the results.

- Our work focuses on date palm species classification, which poses a more fine-grained and challenging task compared to date palm detection and fruit classification. Unlike existing datasets that primarily rely on aerial images, we utilize orchard images to capture more detailed leaf and trunk characteristics. These features, including leaf morphology and trunk attributes such as bark texture, thorns, and leaf scars, play a vital role in distinguishing between different date palm species. Incorporating these important data characteristics into our dataset enhances the accuracy and robustness of our species classification approach.
- Our work introduces a novel self-collected dataset of 2358 images of four common and valuable date palm species (Barhi, Sukkari, Ikhlas, and Saqi). This is the first public dataset of date palm images that covers multiple species. Our dataset can be used as a benchmark for future research on date palm analysis tasks such as gender classification and age estimator.
- Our work demonstrates a robust and practical image-based date palm species classification method using a lightweight and efficient model called *DPXception*. Our model achieved the highest accuracy and inference time compared to other similar proposed approaches. We also developed an Android smartphone application that incorporates our model and can classify date palm species from images captured by the smartphone’s camera in real-time. This provides a convenient and low-cost solution for date growers and others who want to identify date palm species in the field.
- Our work differs from the existing works in several aspects. We do not rely on fruit characteristics, which are not always available or visible, especially in early stages of growth. We do not use robotmounted cameras, which are expensive and complex to operate and maintain. We do not use seedling images, which are not representative of mature date palms and may vary depending on environmental factors. We do not use DNA markers or other sources, which are invasive and time-consuming to obtain and analyze.

## 3 Proposed approach

This section outlines our proposed system pipeline for image-based date palm species classification and its main components. We also introduce the self-collected date palm dataset and the preprocessing techniques we applied before training. Moreover, we explain the proposed *DPXception* architecture. Finally, we discuss the model deployment and demonstrate the development process of the proposed Android application that can accurately classify date palms in real time using a mobile camera.

### 3.1 System pipeline

**Figure 2:**
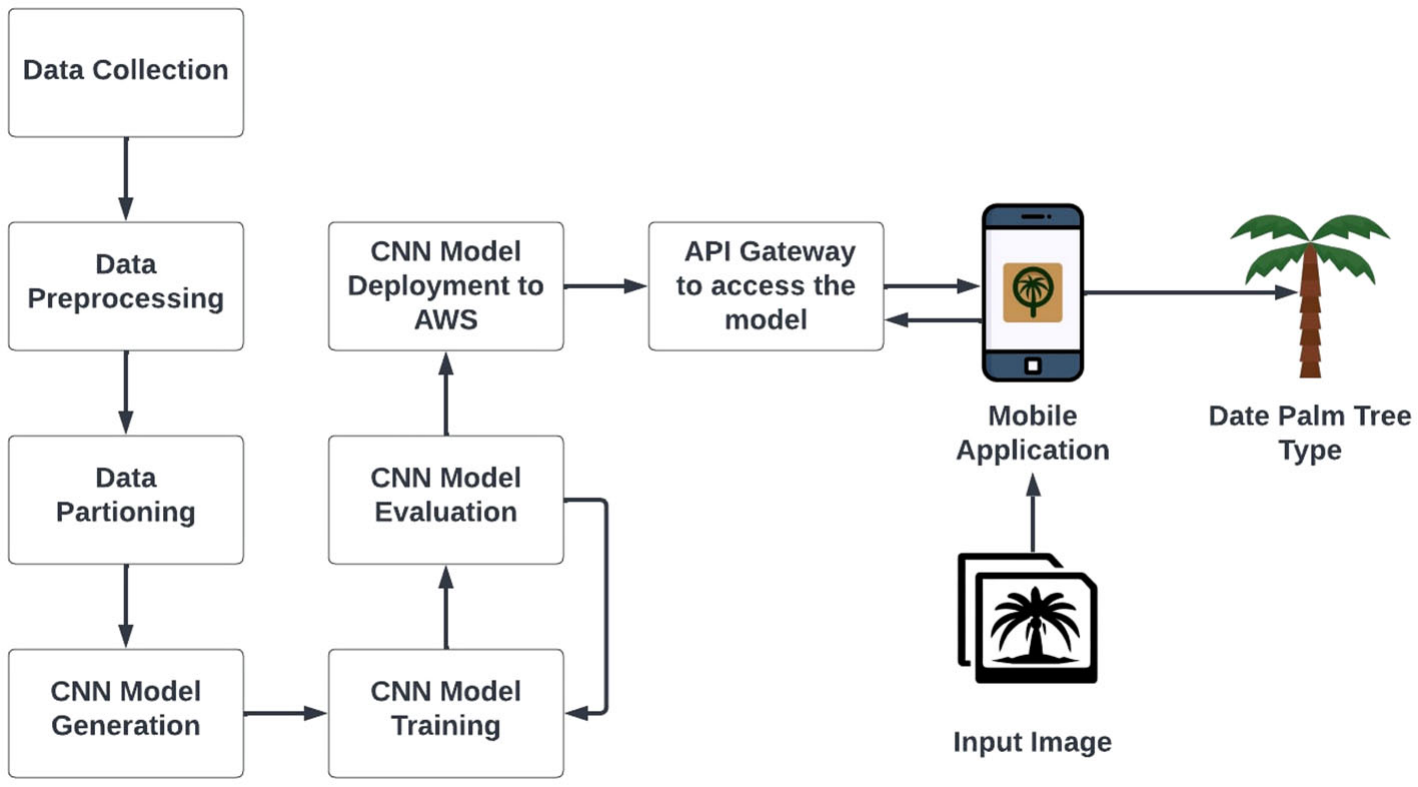
Proposed system pipeline of the image-based date palm species classification.

The proposed system pipeline for image-based date palm species classification is depicted in **Figure 2**. The system consists of several components as follows: (1) date palm dataset creation: we collect and annotate images of four distinct types of date palms to create a comprehensive dataset; (2) data preprocessing: we apply various data augmentation techniques, such as resizing, flipping, and rotation, to enhance the quality of the dataset; (3) data partitioning: we split the dataset into three subsets: training, validation, and testing, ensuring a balanced distribution of date palm types in each subset; (4) CNN model generation: we generate an improved version of Xception CNN architecture named *DPXception* that is tailored for the date palm dataset we constructed; (5) CNN model training with transfer learning: we experiment with well-known CNN models and fine-tune them on our date palm dataset, comparing their performance to *DPXception*; (6) CNN model evaluation: we evaluate the performance of the CNN models on the test subset using various classification metrics, such as accuracy, precision, recall, F1-score, and inference time; (7) CNN model deployment: we deploy the *DPXception* model to the cloud; (8) API establishment: we establish an API gateway to access the *DPXception* model on the cloud and make predictions; and (9) Android application development: we develop and publish an Android application called MouarfAlNakheel, which enables users to upload and classify date palms from their images in real time.

### 3.2 Dataset

**Figure 3:**
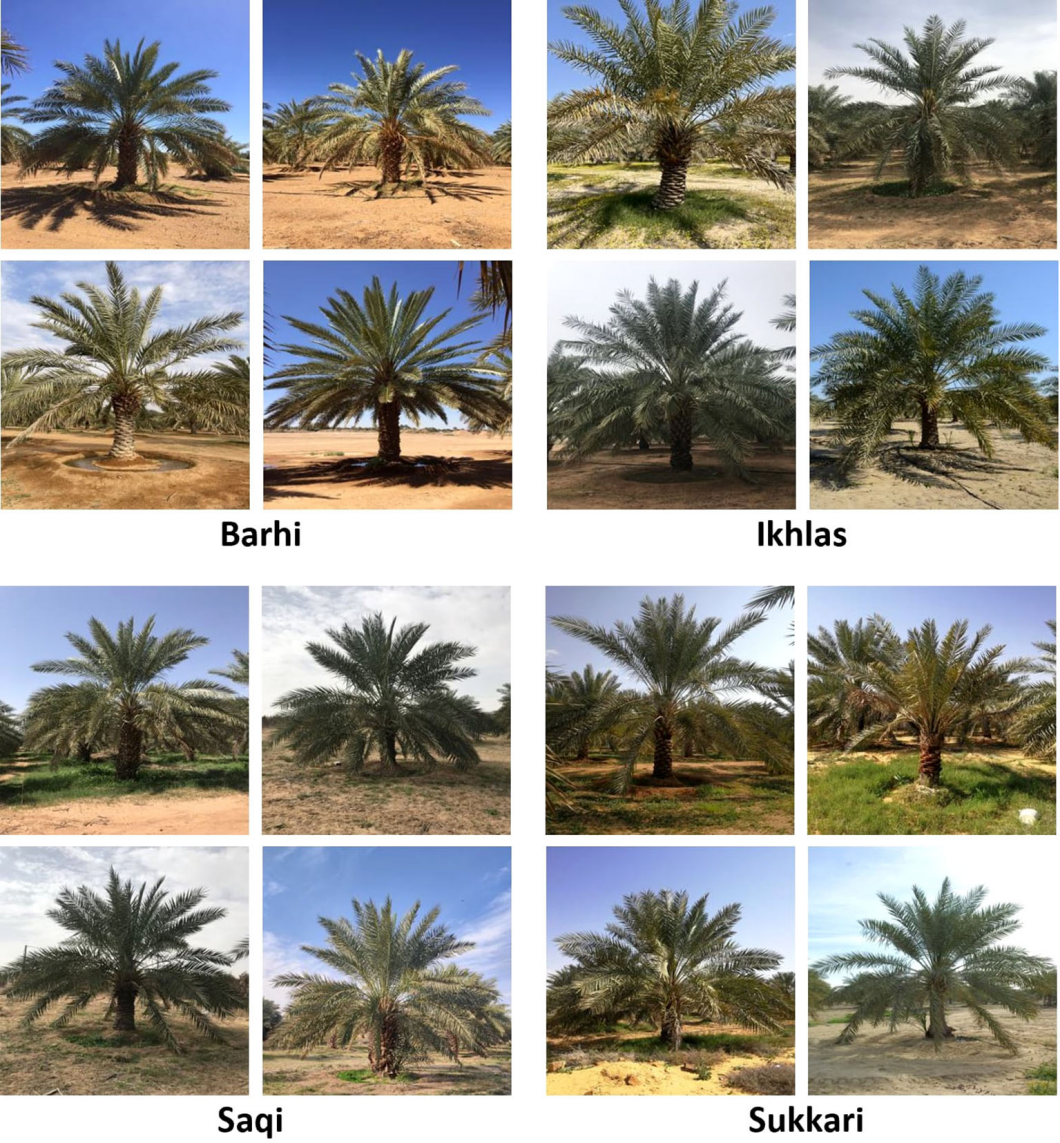
Samples of the four date palm species in the dataset.

We searched for an existing image-based dataset for date palm species classification that could be used to train our model, but we found none. Therefore, we collaborated with the National Center for the Prevention & Control of Plants Pests & Animal Diseases (WEQAA, 2023) to create a novel dataset from scratch. Experts at the WEQAA Center identified the most prevalent date palm species in Saudi Arabia: Barhi, Sukkari, Ikhlas, and Saqi. Our team and voluntary field supervisors from the WEQAA Center then collected and annotated images of these species from farms across different regions of Saudi Arabia. The annotations were verified and validated by experts at the WEQAA Center. We obtained a total of 2358 images: 568 of Barhi, 623 of Sukkari, 635 of Ikhlas, and 523 of Saqi. Samples of the four date palm species are shown in **Figure 3**. We divided the dataset of the date palm species into three subsets as follows: 80% for training (1885 images), 10% for validation (234 images) and 10% for testing (239 images). We used stratified random sampling to ensure a balanced distribution of labels across all the subsets. **Table 1** illustrates the details of the date palm dataset and the label distribution for each subset.

**Table 1:** Date palm dataset details.

Class	Training	Validation	Test	Total Images
Barhi	454	56	58	568
Sukkari	498	62	63	623
Ikhlas	508	63	64	635
Saqi	425	53	54	532
Total	1885	234	239	2358

**Figure 4:**
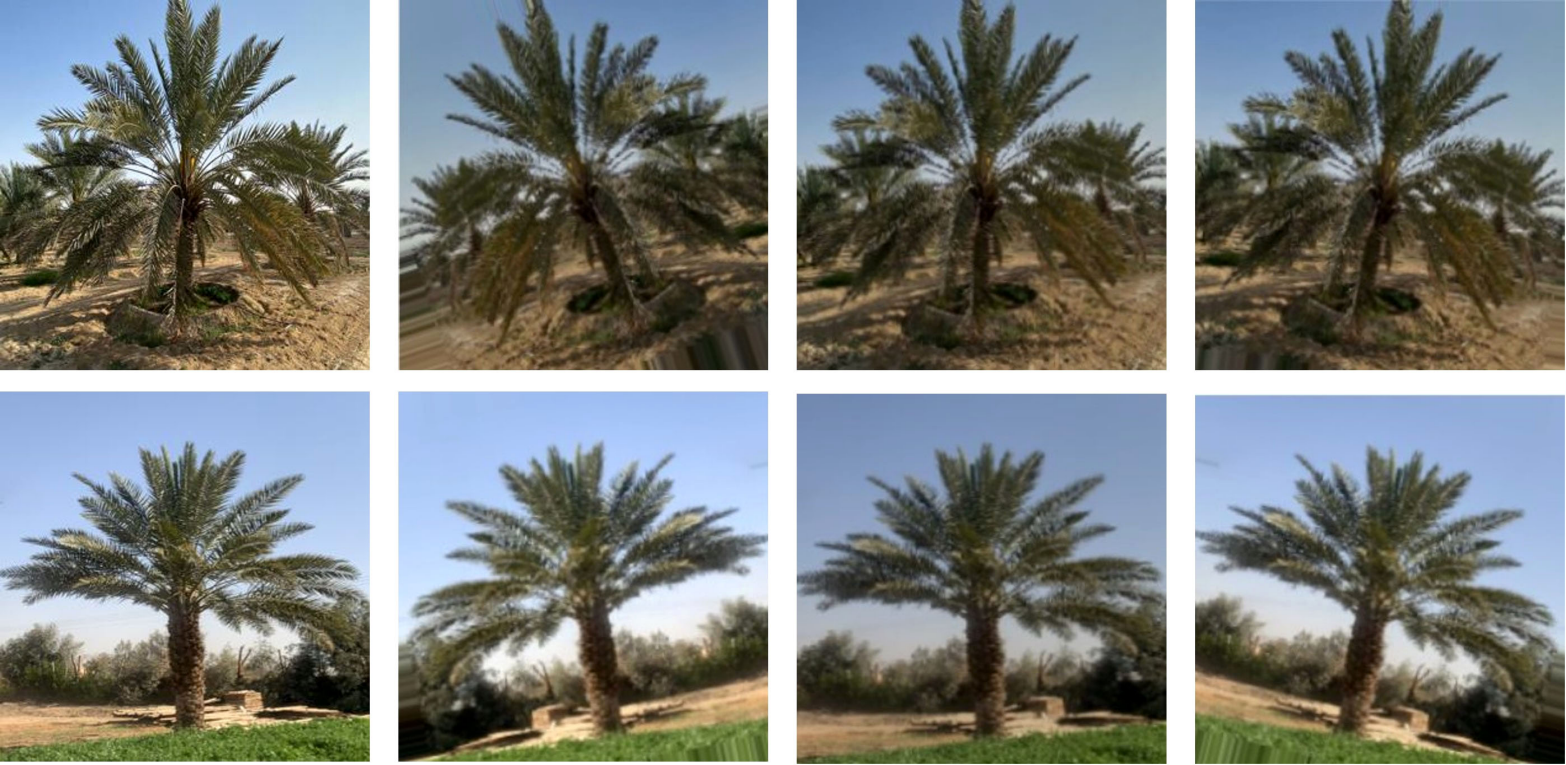
Samples of image augmentation on Ikhlas (first row) and Sukkari (second row).

In this study, we applied data augmentation techniques such as rotation, flipping, and cropping to the images to increase the diversity and generalization ability of the training data. These techniques also helped to prevent overfitting the data. **Figure 4** illustrates some examples of the augmented images. The original images were modified by adjusting the brightness, flipping horizontally, and rotating randomly to create new images.

### 3.3 Proposed CNN architecture

We propose an improved version of Xception, named *DPXception*, specifically tailored for image-based date palm species classification. Unlike the original Xception model, our *DPXception* model utilizes only the first 100 layers of the Xception model for feature extraction, making it more lightweight and efficient. Moreover, our model applies normalization and augmentation techniques prior to the adapted Xception and also reduces the model dimensionality through newly added global average pooling layer after feature extraction.

To help us find a customized model for our dataset, we used Autokeras (Jin et al., 2023), a tool that applies Neural Architecture Search (NAS) to automatically search for the optimal architecture for a model. We set the maximum number of trials to 120 with the aim of finding the model that achieves the highest validation accuracy. Each trial was run for 20 epochs as our primary interest was in evaluating the architecture’s suitability for the dataset.

**Figure 5:**
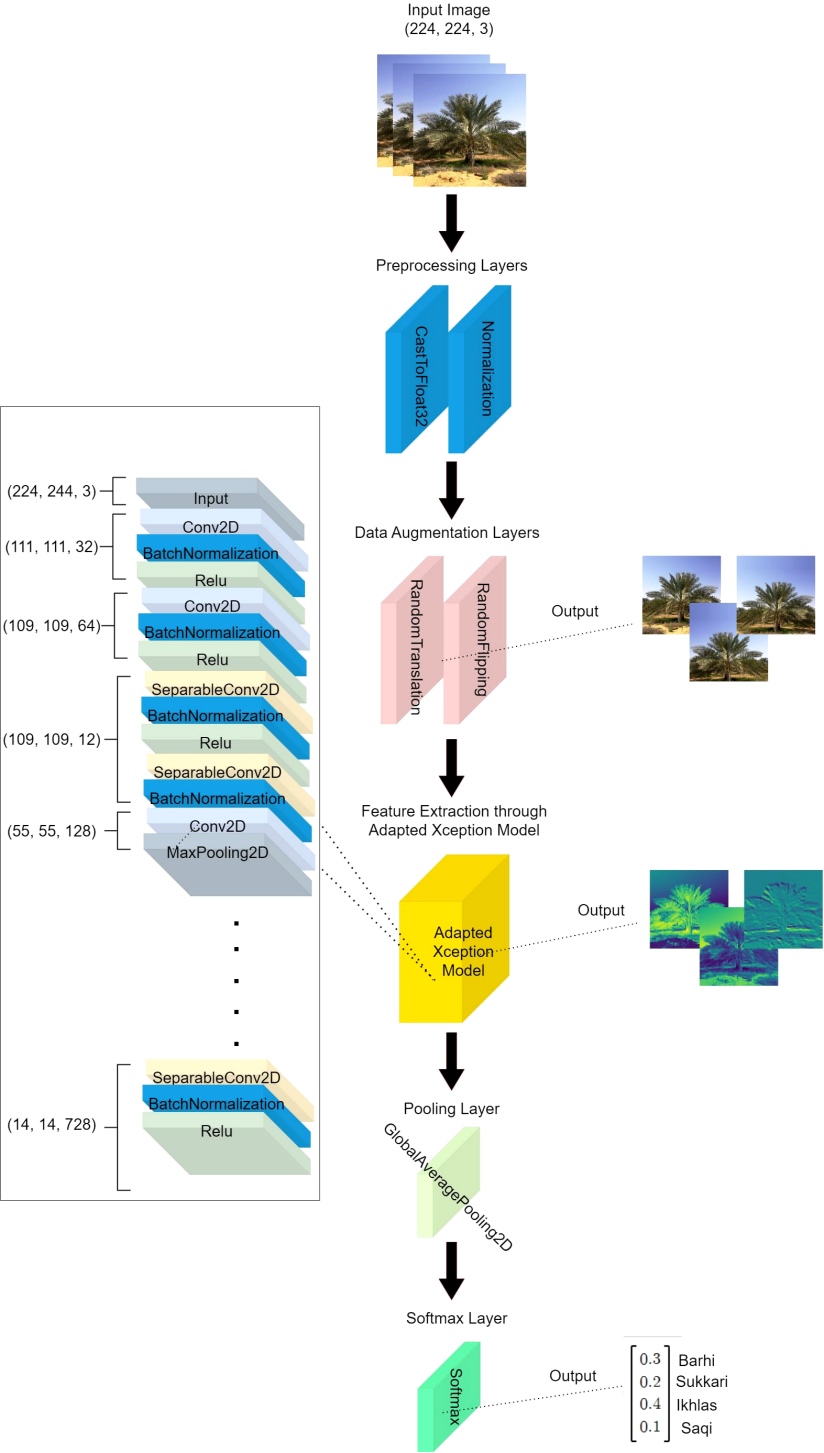
*DPXception* architecture.

As shown in **Figure 5**, the *DPXception* model begins with an input layer that accepts an image of shape (224, 224, 3). This is followed by a CastToFloat32 layer that converts the data type to float32 and a normalization layer that normalizes pixel values to a 0-1 scale. Data augmentation techniques are then applied directly using random translation with 0.1 height and width factors and a random horizontal flipping layer. These techniques enhance the diversity of our dataset and improve our model’s robustness.

Feature extraction is performed by our adapate Xception model, which uses 75% of the original Xception layers - specifically, the first 100 out of 132 layers. It starts with an input layer followed by Conv2D, BatchNormalization, Relu layers with an output shape of (111, 111, 32), and continues through a series of layers until it concludes with a set of SeparableConv2D, BatchNormalization, Relu layers with an output shape of (14, 14, 728). Through experimentation with different sets of layers in Xception and comparison with the original Xception, we found that selecting the first 100 layers resulted in the optimal performance for our dataset.

Following feature extraction, dimensionality is reduced by a Global Average Pooling layer which is a new layer added after the adapted Xception model. A Softmax Layer then maps this 1D feature vector to a 4D vector where each element corresponds to one of the classes in our dataset and applies the softmax function to produce class probabilities. In total, *DPXception* has 11,366,052 parameters, with 11,331,524 being trainable and 34,528 being non-trainable, making it nearly a half of the size of the original Xception model.

### 3.4 Evaluation metrics

We use several evaluation metrics to compare the performance of our proposed *DPXception* model and other CNN models on our date palm dataset. These metrics quantify how well the models can correctly identify the date palm species from images. The following are the evaluation metrics that we use:

Accuracy: This metric measures how many samples were correctly classified by the model. It is calculated as follows:


$$\text{Accuracy}=\frac{\text{True Positives}+\text{True Negatives}}{\text{Total Number of Predictions}}$$

Precision: This metric measures how many of the positive predictions made by the model were actually positive. It is calculated as follows:


$$\text{Precision}=\frac{\text{True Positives}}{\text{True Positives}+\text{False Positives}}$$

Recall: This metric measures how many of the actual positive samples were correctly predicted by the model. It is calculated as follows:


$$\text{Recall}=\frac{\text{True Positives}}{\text{True Positives}+\text{False Negatives}}$$

F1-score: This metric provides a balanced measure of the model’s performance by combining precision and recall. It is calculated as follows:


$$\text{F}1=2\times \frac{\text{Precision}\times \text{Recall}}{\text{Precision}+\text{Recall}}$$

Inference Time: This metric measures the time it takes for the model to make a prediction on new data. It is usually measured in milliseconds or seconds and can vary depending on the complexity of the model, the size of the input data, and the hardware used for inference.

### 3.5 Model deployment

After selecting the best-performing model, we deployed it to Amazon Web Services (AWS) (AmazonWeb-Services, 2023), a cloud computing platform offered by Amazon. To deploy our model, we first stored it in an S3 bucket, a scalable object storage service provided by AWS. We then created an endpoint to access the model stored in the S3 bucket.

Next, we connected the model to a Lambda function, a serverless computing service provided by AWS. The Lambda function takes an input image, resizes it to the appropriate dimensions and passes it to the model to generate a prediction. The prediction is then returned to the user as the output of the Lambda function.

To facilitate access to the model from our Android application (will be discussed next), we established an API gateway, a fully managed service provided by AWS that enables developers to create and manage APIs allowing us to easily interact with the Lambda function from our Android application.

### 3.6 The date palm identifier app

**Figure 6:**
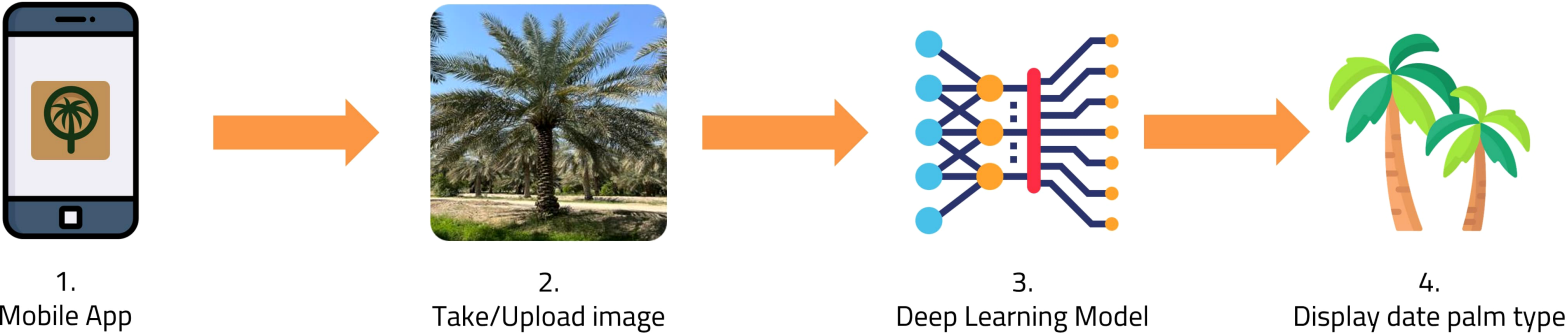
MouarfAlNakheel workflow.

**Figure 7:**
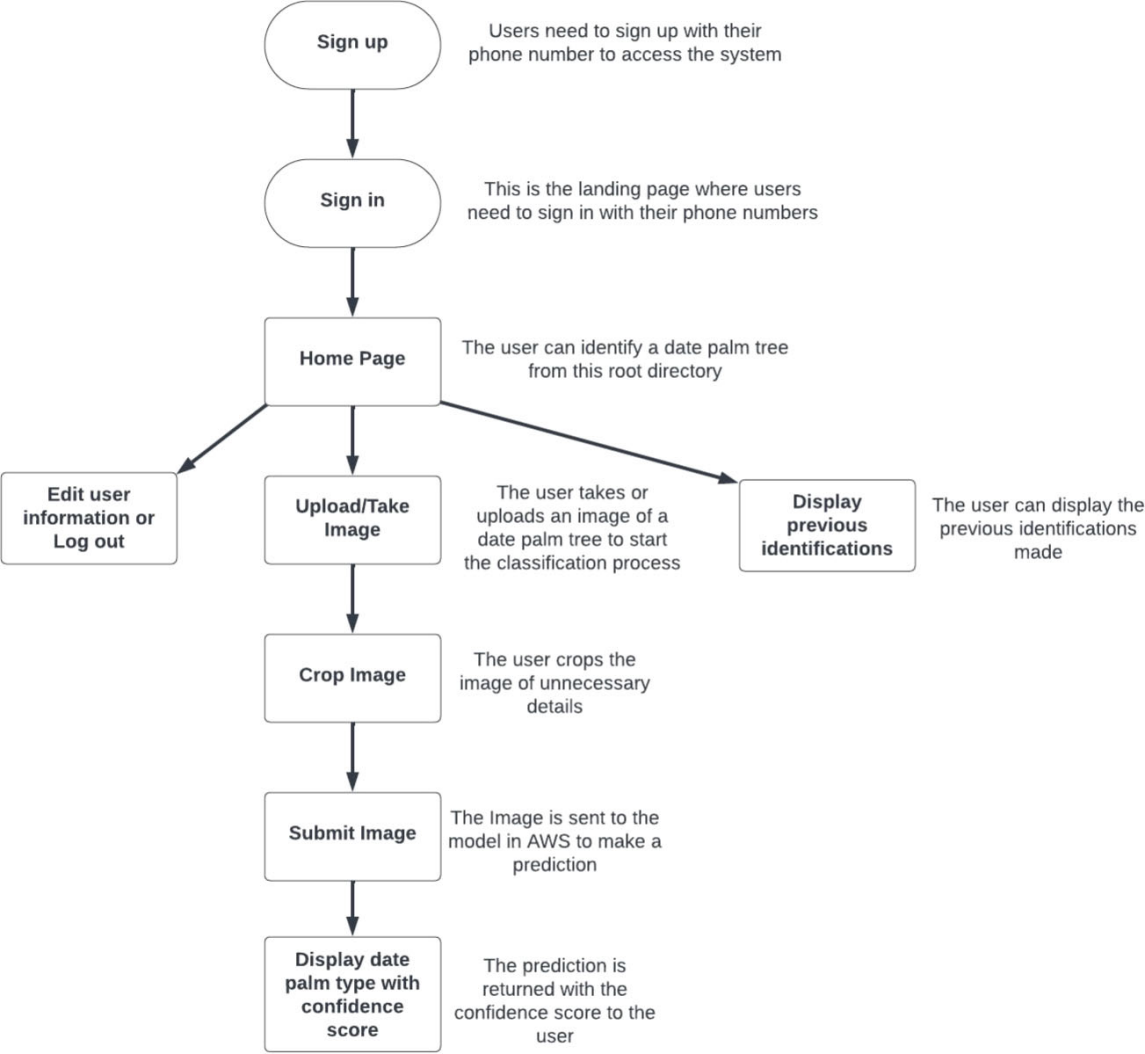
The development stages of the MouarfAlNakheel app for image-based date palm classification.

**Figure 8:**
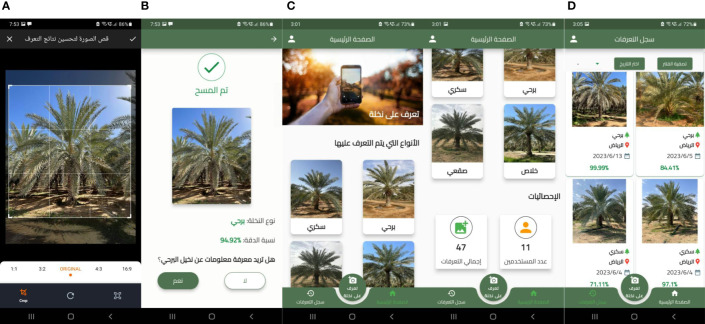
The MouarfAlNakheel Screenshots where **(A)** is the new classification page, **(B)** is the results page, **(C)** is the home page, and **(D)** is the history page.

To utilize the model we deployed to the cloud, we incorporated it into an Android mobile application named MouarfAlNakheel^1^. The main workflow of MouarfAlNakheel is illustrated in **Figure 6**. The development stages of MouarfAlNakheel app for image-based date palm classification are illustrated in **Figure 7**. MouarfAlNakheel allows users to take or upload an image of a date palm tree using their mobile phones, and then crop the image to remove unnecessary details (as depicted in **Figure 8A**). The application sends the cropped image to the deployed model on AWS to make a prediction, and then returns the date palm tree type along with the confidence score to the user as well as an option to view general information of the predicted type (as depicted in **Figure 8B**). The application also provides general information about the four date palm types on its home page (as depicted in **Figure 8C**), as well as displaying the total number of identifications or predictions made by users and the total number of users. Additionally, the application displays previous identifications made by the user on its history page (as depicted in **Figure 8D**), specifying the date palm type, location where the image was taken or uploaded, date of identification, and confidence score. The history page also includes a filter that allows users to select a specific date palm type or date, and then displays only the previous identifications of that type or date.

## 4 Results and discussion

This section reports the experimental results of *DPXception* model and compares it with seven representative CNN models that are trained on our date palm dataset. We first present the experimental setup, including the hardware and software specifications, and explain the hyperparameters used to train *DPXception* and other CNNs. Then, we evaluate and compare the performance of *DPXception* with selected alternative CNN models. Finally, we investigate the impact of data augmentation on enhancing the effectiveness of *DPXception* model.

### 4.1 Experimental setup

To implement and generate our convolutional neural networks, we used the Python programming language and Google’s TensorFlow framework (TensorFlow, 2023), an open-source platform for machine learning, deep learning, and other data-driven workloads. We conducted our experiments on Jupyter Notebook, using the TensorFlow 2.13.0 library to create artificial neural networks and a NVIDIA GeForce GTX 1660 SUPER GPU to speed up training. We obtained all models used in this experiment from TensorFlow Hub (TensorFlowHub, 2023).

### 4.2 CNNs and hyperparameter settings

To compare the performance of our *DPXception* model with other models, we trained seven representative models of similar size on our date palm species dataset. The selected models are Xception, ResNet50, ResNet50V2, InceptionV3, DenseNet201, EfficientNetB4, and EfficientNetV2-S. We used the Adam optimizer with learning rate 0.0001 for all experiments, as it outperformed other optimizers such as Stochastic Gradient Descent (SGD) in our preliminary tests. The batch size was set to 16 for all models and the learnable parameters were initialized using Keras’ default values. We used categorical crossentropy as the loss function, as it is suitable for multi-class classification problems with mutually exclusive classes. We did not use any dropout layers or regularization layers as they did not improve our results, possibly because our dataset is relatively large and balanced. We evaluated the models using both loss and accuracy metrics. We trained all models for 100 epochs, but we used an early stopping callback function that monitors the validation loss and saves the model based on the minimum validation loss during training. The early stopping function has a patience of 10, which means that it stops the training if the validation loss does not improve for 10 consecutive epochs. The hyperparameters for the experimented CNNs are shown in **Table 2**.

**Table 2:** Hyperparameter values.

Training Component	Approach and Values
Number of epochs	100
Batch size	16
Learning rate	0.0001
Loss function	Categorical Crossentropy
Weight decay	None
Optimizer	Adam
Regularization	None
Dropout	None

### 4.3 Experimental results

We evaluated the performance of our proposed model, *DPXception*, on our date palm dataset. We compared it with seven other models based on their accuracy, F1 score, inference time, and model size. We also analyzed the accuracy and loss plots, the confusion matrix, and the class-wise metrics of our model.

#### 4.3.1 Model comparison

**Table 3** summarizes the results of the comparison in terms of accuracy, F1-score, and model size. *DPXception* outperforms the other models, achieving the highest accuracy and F1-score of 92.9% and 93%, respectively. Xception and InceptionV3 are the second most effective models, with identical accuracy and F1-score of 92.4% and 93%, respectively. EfficientNetB4 ranks the third, with an accuracy and F1-score of 91.6% and 92%, respectively. ResNet50V2 and DenseNet201 have comparable performance, with an accuracy and F1-score of 90.3% and 91%, respectively. ResNet50 is the fourth most effective model, with an accuracy and F1-score of 89.9% and 90%, respectively. EfficientNetV2-S is the least effective model, with the lowest accuracy and F1-score of 88.2% and 88%, respectively.

**Table 3:** Comparison of performance metrics of the CNN models on the test dataset.

Model	Accuracy	F1-score	Size
Xception	92.4%	93%	22.9M
ResNet50	89.9%	90%	25.6M
ResNet50V2	90.3%	91%	25.6M
InceptionV3	92.4%	93%	23.9M
DenseNet201	90.3%	91%	20.2M
EfficientNetB4	91.6%	92%	19.5M
EfficientNetV2-S	88.2%	88%	21.6M
**DPXception**	**92.9%**	**93%**	11.4M

The bold values highlight our proposed approach as it outperforms other approaches in terms of accuracy.

**Table 4** summarizes the average inference time of each model in seconds on both GPU and CPU devices. *DPXception* is the fastest model on both GPU and CPU platforms, with an inference time of 0.0513 seconds and 0.1175 seconds, respectively. Xception is the second fastest model on GPU, with an inference time of 0.0799 seconds, while ResNet50V2 is the second fastest model on CPU, with an inference time of 0.1614 seconds. DenseNet201 is the slowest model on both GPU and CPU platforms, with an inference time of 0.2085 seconds and 0.4705 seconds, respectively. These results demonstrate that our *DPXception* model not only achieved the highest accuracy and F1-score, but also the lowest inference time among all compared models.

**Table 4:** Comparison of inference time of the CNN models.

Model	GPU (s)	CPU (s)
Xception	0.0799	0.1700
ResNet50	0.0891	0.1815
ResNet50V2	0.0847	0.1614
InceptionV3	0.1080	0.2200
DenseNet201	0.2085	0.4705
EfficientNetB4	0.1827	0.4029
EfficientNetV2-S	0.1899	0.4233
**DPXception**	**0.0513**	**0.1175**

The bold values highlight our proposed approach as it outperforms other approaches in terms of time efficiency.

#### 4.3.2 Model analysis

**Figure 9:**
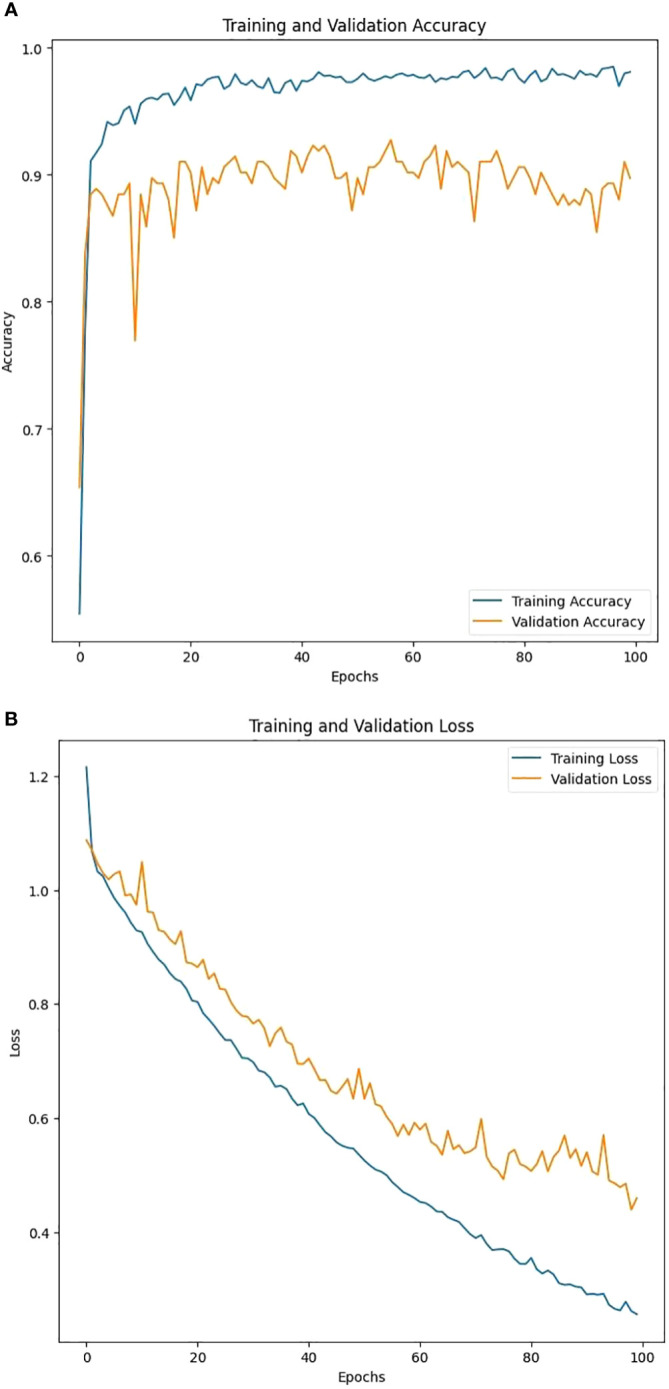
Training and validation behaviors across the 100 epochs for *DPXception* where **(A)** represents the training and validation accuracy and **(B)** represents the training and validation loss.

**Figure 9** shows the accuracy and loss plots of our *DPXception* model during training and validation. The plots indicate that the model performs well in accurately classifying data, with both training and validation accuracy increasing over time. Early stopping was employed during training to prevent overfitting, and while there are some fluctuations in the validation loss, it is possible that overfitting may not be a significant issue.

**Figure 10:**
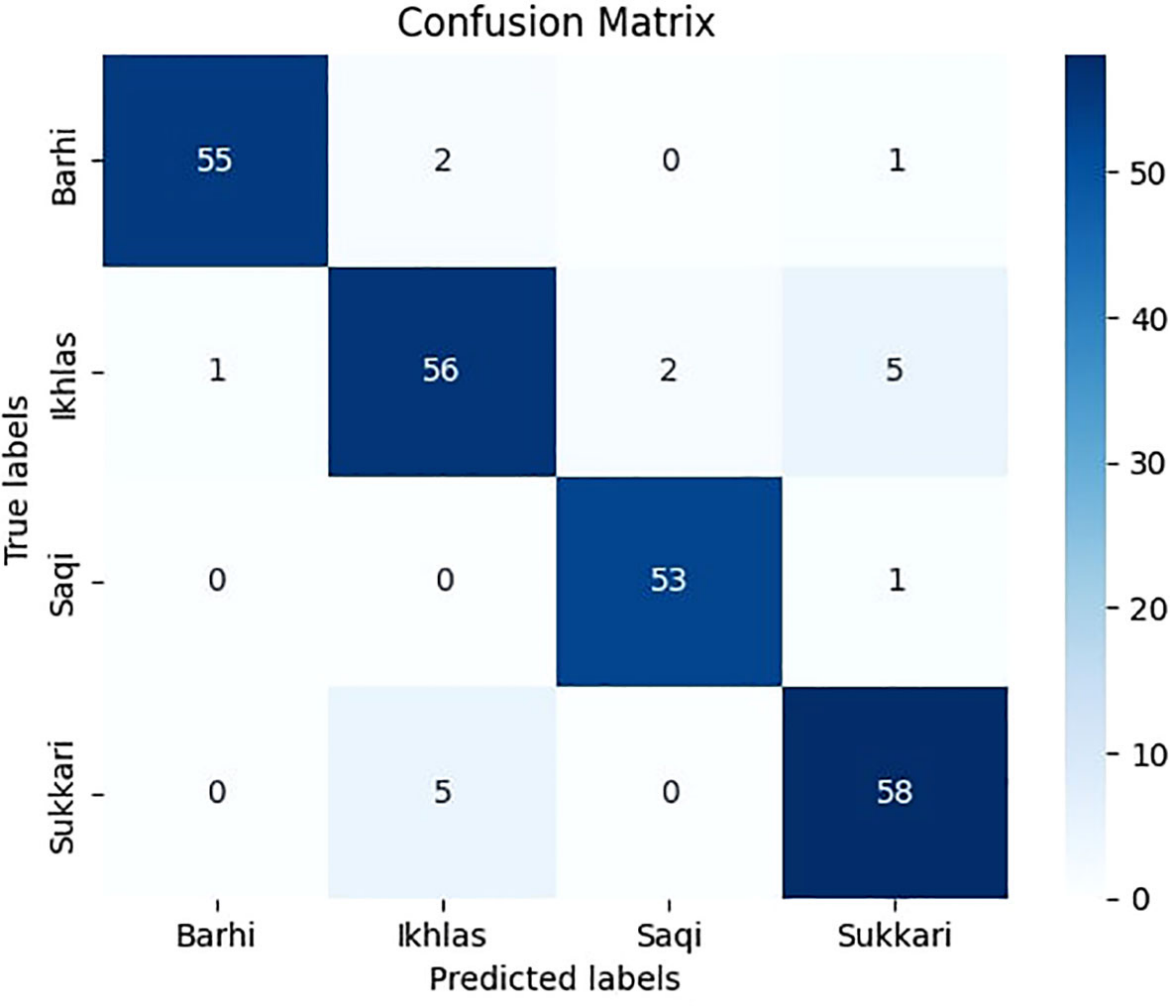
Confusion matrix of the *DPXception* model.

**Figure 10** shows the confusion matrix of our *DPXception* model on the test dataset. The confusion matrix summarizes the number of correct and incorrect predictions for each class. Additionally, **Table 5** displays the precision, recall, and F1 score for each class of *DPXception*. As shown in **Table 5** and **Figure 10**, the classes with the lowest F1 scores (88% and 91%) are Ikhlas and Sukkari, respectively. This is due to the misclassification of 5 Ikhlas images as Sukkari and 5 Sukkari images as Ikhlas by the *DPXception* model. This is likely because these two classes of date palm trees have similar features such as leaf brightness and shapes.

**Table 5:** Accuracies of *DPXception* for each class on test set.

Class	Precision	Recall	F1-score
Barhi	98%	95%	96%
Sukkari	89%	92%	91%
khlas	89%	88%	88%
Saqi	96%	98%	97%
Average	93%	93%	93%

Our model differs from the original Xception model in several aspects. Firstly, our model uses only the first 100 layers of Xception, making it more lightweight and efficient. Secondly, our model applies normalization and augmentation prior to adapted Xception, which improves the accuracy and robustness of our model. Thirdly, our model reduces the dimensionality by adding an extra global average pooling layer after feature extraction by adapted Xception, which affects the performance and interpretability of our model. These design decisions are the potential causes that lead to our model’s outperformance over the original Xception model and other contrastive models in terms of accuracy, F1-score, and inference time.

### 4.4 Effect of data augmentation

Data augmentation is a common and useful technique in deep learning, especially when dealing with small or imbalanced datasets. We experimented with several data augmentation techniques, including random rotation, random flipping, random brightness, and random translation. Each of these techniques contributed to improving the performance of our model by increasing the diversity of the training data and reducing overfitting. By introducing variations in rotation, flipping, brightness, and translation, our model was able to learn more robust features that are invariant to these transformations, resulting in better generalization to new images.

The results showed that data augmentation significantly improved the accuracy of our model from 89% to 92.9%, indicating that it can generalize well to new images. It is important to note that the same data augmentation has been applied to all the models experimented in this work, and here we only show the effect of data augmentation on our model. Similar effects were observed with all other models experimented – data augmentation does help all the models achieve better accuracy and robustness.

## 5 Conclusion and future work

In this paper, we have proposed a novel method for image-based date palm species classification using a lightweight and efficient CNN model called *DPXception*. We have also introduced a new dataset of 2358 images of four common and valuable date palm species. To increase the size and diversity of our dataset, we applied data augmentation techniques. Our model differs from the original Xception model in several aspects. Firstly, our model uses only the first 100 layers of Xception (resulted in an adapted Xception) for feature extraction, making it more lightweight and efficient. Secondly, our model applies normalization prior to adapted Xception, which improves the accuracy and robustness of our model. Thirdly, our model reduces the dimensionality by adding an extra global average pooling layer after feature extraction, which affects the performance and interpretability of our model. Our experimental results show that our model outperforms the original Xception model and other contrastive models in terms of accuracy, F1-score, and inference time. We have also demonstrated the practical applicability of our model by developing a smartphone application that can classify date palm species in real-time from the camera. To the best of our knowledge, this is the first work to provide a public dataset of date palm images and to show a robust and practical image-based date palm species classification method. This work will pave the way for more advanced date palm analysis tasks such as gender and age estimation.

For future work, we plan to extend our method to other date palm species and to other types of date palm images. We also intend to explore the use of our model for other date palm analysis tasks, such as gender and age estimation, disease detection, and yield prediction. We hope that our work will inspire more research on image-based date palm analysis and contribute to the advancement of date palm agriculture and economy.

## Data availability statement

The raw data supporting the conclusions of this article will be made available by the authors, without undue reservation.

## Author contributions

MS: Conceptualization, Formal analysis, Funding acquisition, Investigation, Methodology, Project administration, Resources, Supervision, Validation, Visualization, Writing – original draft, Writing – review & editing. WA: Data curation, Formal analysis, Methodology, Software, Validation, Visualization, Writing – review & editing. SA: Data curation, Funding acquisition, Project administration, Resources, Supervision, Validation, Visualization, Writing – review & editing.

## Footnotes

1
https://play.google.com/store/apps/details?id=com.DPTIDAPP.flutter_application_1
